# Does widowhood affect social capital in old age: the case of India

**DOI:** 10.3389/fsoc.2025.1549004

**Published:** 2025-06-09

**Authors:** Akanksha Choudhary, Ashish Singh, Mohammad Hifz Ur Rahman

**Affiliations:** ^1^School of Liberal Arts, Indian Institute of Technology Jodhpur, Jodhpur, India; ^2^SJM School of Management, Indian Institute of Technology Bombay, Mumbai, Maharashtra, India; ^3^Public Health, College of Medicine and Health Sciences, National University of Science and Technology, Sohar, Oman

**Keywords:** widowhood, women, old age, social capital, sociability, India

## Abstract

**Purpose:**

In India, widowhood, besides, being a personal status, presents itself as a social institution. Widows in India have long been deprived of normal living conditions and their hardships have been generally inconspicuous to policymakers as well as researchers. Therefore, this study is an attempt to understand the social dynamics of widowhood in India mainly through empirical means. It aims to examine the social capital of old widows through the lens of sociability, safety, and trust and solidarity.

**Methods:**

Ordered logistic regressions and Item Response Theory Partial Credit models with data drawn from the SAGE Study on Global Aging and Adult Health which was conducted across six populous states of India have been used for analysis.

**Findings:**

Results show that old widows experience significantly lesser sociability, trust and solidarity as compared to their married counterparts. Also, older widows from high income group are less vulnerable and have higher social capital compared to that of older widows from poorer income groups.

**Discussion:**

Policy implications drawn based on the findings can play a vital role in uplifting the life of old widows in India significantly. This study is perhaps the first study to capture how widowhood affects the social capital in old age in India. Besides, based on the findings, we also suggest some policy initiatives to address the concern of low social capital among old widows in India.

## Introduction

“*The death of a woman‘s husband marked the transition from wife to widow taking the woman from a central place in the family to its margin; henceforth she was regarded as someone who was physically alive but socially dead.”*

–*Chakravarti (**1995**)*

From an early age, life is a struggle for girls in most parts of the developing world. Women's equality in terms of education, employment, and bargaining power at home is still an individual rather than a universal achievement (Choudhary et al., 2019). For most of the things, such as education, inheritance of property, freedom to make decisions, etc., which are served on the platter to the males of the households, females have to struggle. The majority of the women in the developing world, are still content with this kind of status which is generally inferior to that of the men (Chen, 1997, 2000; Choudhary and Singh, 2017, 2019, 2024). Talking about social status, a woman typically goes through majorly three phases in her life, namely as a daughter, wife, and mother. Between a wife and a single woman, it is the wife who enjoys a higher social status. Marriage brings social acceptability to women, but all of this takes a downturn when a woman loses her partner or husband (Chen, 1997, 2000). The loss of a partner in itself is devastating, and for many women, that loss gets accompanied with an everlasting struggle for basic needs, human rights and dignity (Chen, 1997, 2000). They are disgraced for life, shunned and shamed. On top of it, most of these abuses go unnoticed and have got social acceptability in many societies (United Nations, 2001). The destitution of widows has for too long been by and large neglected and living condition of many widows remains more or less deplorable. This not only hampers a country socio-economically but also qualifies as a humanitarian emergency that needs to be addressed as a significant human rights violation (The Global Widows Report, 2015).

This issue becomes even more critical when we look at the population of widows around the world which is estimated to be about 258 million. Nearly one in ten out of these, lives in extreme poverty (United Nations, 2001). They have to go through other hardships in their life such as starvation, vulnerable to physical and sexual assaults, falling prey to diseases such HIV/AIDS, seizure of their homes and other possessions as well as social exclusion. They too have normal needs like any other woman, but their voices and sufferings are often absent from policies that impact their survival (United Nations, 2001). Across the globe, the status of widows in different societies and cultures has varied with time.

The condition of widows has improved over time in some of the societies, while in others; it has declined or remained mostly unchanged. One such country where the social status of widows has mostly remained unchanged over time is India. The condition and status of widows in India is quite complex and varies with the region as well as religion. Interestingly, India is the only country where widowhood, besides, being a personal status, presents itself as a social institution (Dreze, 1990). Widow's stigmatization and deprivation are made worse by cultural symbolism and rituals (Dreze, 1990). Indian society, like many other patriarchal societies, deliberates the social status of a woman through a man; wherein, in the absence of a male partner, she becomes a non-entity, ultimately suffering a ‘social death' (Dreze and Srinivasan, 1997).

In India there is not only a dearth of policies and government intervention to help Indian widows to lead a normal life but also only a minuscule amount of attention has been paid by academicians and researchers to address their plight. Given the above, this study makes an attempt to understand the social dynamics of widowhood in India mainly through empirical means. The study analyses the bonds of older widows with the society and its members through different components of social capital, namely sociability, safety, trust and solidarity. This helps in understanding and addressing the barriers which negatively impact the social capital of the older widows in India. Before entering into an empirical investigation of the social status of Indian widows, it is crucial to throw some light on the deep-rooted customs (for the Indian widows) derived from the ancient socio-religious scriptures.

### Historical and religious perspective of widowhood in India

It has been argued that women in India relished equal status and rights during the early Vedic period (c. 1500 to c. 1200 BCE).^1^ However, later (approximately 500 BCE), the status of women began to deteriorate with the advent of ‘Smritis (esp. Manusmriti)'^2^ (Rani, 2017). Whenever there is a discussion about widowhood in India, the phenomena which garner attention are related to *Sati*, widow remarriage, inheritance and traditions related to dress, tonsure and food among widows from the beginning of Vedic times (Altekar, 1956; Rani, 2017; Lamb, 2000; Mani, 1998). Widowhood was often treated as a punishment by God for some sins done in the previous births and these beliefs came from The ‘Brahmanical Authoritarian'^3^ texts, like the Dharma Shastras and Dharma Sutras (c. CE.100–CE.500).

The situation in the post Vedic era did not get any better and the deterioration of their condition continued with time and especially it became worse during the Mughal era (1526–1857), where women's freedom and rights were further curtailed (Rani, 2017). However, during the British rule in India (1858–1947), British administrators tried to improve the situation of widows by introducing inheritance laws for widows and the prohibition of *Sati*. Unfortunately, as a result of that, the tradition of deporting widows to holy cities became rampant and with that, the concept of abandoned widows emerged (Nayar, 2006). Families with more impoverished socio-economic conditions in the northern parts of India started dispatching the widows as mere objects to the holy cities of northern India, like Mathura, Varanasi, and Vrindavan. This was done under the pretence that they should pass the rest of their lives in religious quests (United Nations, 2001). These abandoned widows not only lived in pitiable conditions but also had to go through sexual and economic exploitation (Jalali, 1994). In these holy cities, young widows were (and are) forced into unholy prostitution, while older ones were (and are) forced to beg from tourists and pilgrims (United Nations, 2001). Nevertheless, it was reported that abandoned widows even considered this life better than the life they would have lived with their family members where practices, traditions and social constraints enacted severe limits on their dress and diet, exploited them and led to social isolation (JISC, 2009). Therefore, widows in India face extreme deprivation, including social exclusion, economic hardship, and vulnerability to violence and exploitation (Niswade, 2015; Chandra, 2011). Widowhood was also associated with a decrease in days worked for older women highlighting the economic vulnerability of women experiencing widowhood in older age (Reed, 2020). Moreover, social stigma and cultural norms often dictate that a widow loses her social identity after the death of her husband, resulting in social ostracization, dispossession of property, and limited economic opportunities. Many widows are left without financial security and are vulnerable to physical and sexual violence, with little protection from society or the state (Chen and Dreze, 1995). The absence of strong social networks exacerbates these issues, leaving them isolated and struggling to access basic needs. Besides, recently widowed women and long-term widows exhibited higher psychological distress, poorer self-rated health, and increased hypertension risk, even after adjusting for covariates, compared to married women in India (Perkins et al., 2016). The scholarship on condition of widows from other parts of the world indicates that the situation of widows is not as miserable as Indian widows. However, they too do go through some discrimination (United Nations, 2001).

Moving on to social capital attached to older widows in India, it is essential first to understand social capital itself.

### Social capital and widowhood

The term ‘social capital' is the central concept for this study, crucial for understanding individual wellbeing and social dynamics. Scholarship in the area started to develop with the contributions of scholars such as Pierre Bourdieu, James Coleman, and Robert Putnam (Bourdieu, 2018; Putnam, 2000; Coleman, 1988). Bourdieu, in his work during the 1970s and 1980s, conceptualized social capital as the aggregate of actual or potential resources linked to possession of a durable network of more or less institutionalized relationships of mutual acquaintance and recognition. His perspective emphasizes the role of social capital in social stratification and the reproduction of inequality (Bourdieu, 2011). Coleman (1988) offered a functional definition, viewing social capital as aspects of social structure that facilitate certain actions of individuals who are within the structure. He identified various forms of social capital, including obligations and expectations, information channels, and social norms, underscoring the importance of closure within social networks for fostering trust and effective sanctions (Coleman, 1988). Putnam (1993) further popularized the concept, defining social capital through its components: moral obligations and norms, social values (especially trust), and social networks (especially voluntary associations). He argued for its significance in the functioning of democratic institutions and the prosperity of economies (Putnam, 1993). The work around social capital can also be often traced to Durkheim (1964), which defined it, as the interdependence between people of society with collective fidelity and integration. Besides, Hanifan's work defines social capital way early in 1916 in a figurative sense as the good-will, fellowship, mutual sympathy, and social intercourse among individuals and families within a social unit, specifically the rural community centered around the school. He argued that an accumulation of this social capital is essential for constructive work in community building (Hanifan, 1916). Beyond these foundational perspectives, Lin (1999, 2000) defined social capital as investment in social relations with expected returns in the marketplace, emphasizing the importance of network resources and structural location. Burt (1998) highlighted the advantages derived from structural holes in social networks, which provide opportunities for brokerage and information access (Burt, 1998). Additionally, the World Bank's report played a key role in disseminating the concept within development economics, linking social capital to various developmental outcomes (World Bank, 2000). Fafchamps (2013) approached social capital by analyzing how personalized and generalized (i.e. society-wide) trust, clubs and social networks of human. Robison and Siles (2002) introduce the notion of socio-emotional goods and attachment values to the more conventional social capital repertoire of networks, institutions and power. Social capital facilitates exchange and is seen as the most effective means of internalizing externalities. Additionally with time, social capital was also perceived as the eagerness of people to work together at all layers of society to accomplish the common goals of welfare and well-being (Jeannotte et al., 2002). Hence, it is also associated with a sense of belongingness and collective identity (Rahman and Singh, 2019). In a nutshell, social capital includes the components, such as ‘sociability', ‘safety', ‘trust and solidarity' that bring and hold individuals together in society and aim to reduce the allied risk factors varying from social wellbeing to health.

That said, there is some apprehension concerning the increased number of widows and associated consequences, such as lack of identity, decrease in the degree of participation in social activities and diminished social recognition all at the same time (United Nations, 2001; The Global Widows Report, 2015Social cohesion becomes even more crucial for the older widows as they have higher chances of experiencing seclusion. The well-being of elderly widows is not just a question of economic security, but also one of dignity, self-respect and participation in society. Many widows suffer from different forms of social isolation, psychological abuse, or emotional distress arising from the perceived threats to the social order and claim of widows on ancestral property. Studies found that social support can resume the pre widowhood status of women (Avis et al., 1991). Therefore, social support has the potential to become a critical factor in averting the vulnerability of widows at older ages. Numerous studies have been conducted about the life and status of widows across the globe. However, no substantial empirical work on the social status of widows has been conducted in the Indian context despite the country having the largest population of widows. Some studies which have examined the social condition of Indian widows date back to the 1990s (Chen, 1997; Dreze, 1990; Dreze and Srinivasan, 1997). Also, these studies were mostly exploratory in nature based on small samples. The existing narrative on the widowhood in India has primarily focussed on health issues, living arrangements, economic status, widow immolation and abandonment. Besides, a large part of the existing scholarship on the widowed population deals with the widows living in shelter homes or the widows who have been abandoned by their family members in holy cities.

To the best of our search, we could not find a single study that has examined the social capital of the widowed population in India on a large scale (across different states) or by using an empirical method. However there have been region focused relevant studies trying to capture social capital through women empowerment in context of widowhood whose findings are of paramount importance in the national context. On the similar lines, Janssens (2010), investigates the impact of a women's empowerment program (Mahila Samakhya) in Bihar on trust and cooperation within rural communities. Findings from the natural experiment study suggested significant increase in localized trust and generalized trust in program villages in comparison to control villages with also some spillover in non participating households from program villages (Janssens, 2010). Given that, now it has been around three decades of high economic growth in India, it will be interesting to see whether the effects of high economic growth have borne any fruit in terms of improved social capital of the widows in India. Also, the present study focusses specifically on older widows (more than 50 years old; 50+) because in the near future the proportion of older adults as a fraction of total population is going to increase exceptionally and their chances of remarrying are minute compared to the widows of relatively younger age (Stevens, 1995; Niswade, 2015). Moreover, this study includes older widows from different regions of India through six populous states, namely Assam, Karnataka, Maharashtra, Rajasthan, Uttar Pradesh and West Bengal. In addition to the consideration of geographic range and population size, these states are at different stages of the demographic and economic development as well as epidemiological transitions. Therefore, our study not only complements the existing evidence on the topic but also adds new insights to the existing scholarship on the subject.

## Data and methods

### Data

The study utilized data from Wave1 of the SAGE Study on Global Aging and Adult Health, conducted across six populous states in India between 2007 and 08. SAGE aimed to provide valid and reliable information on the wellbeing and health outcomes of the geriatric population, to better understand the effects of aging, economic and social changes, policy changes, and healthcare on current and future health (He et al., 2012). The same data set was also used to study the importance of social capital for healthy aging in Indian context (Himanshu et al., 2019). The study used multistage cluster sampling and categorized households into “50+ households” and “18–49 households” (Kowal et al., 2012). All individuals aged 50 years and above from “50+ households” were selected, while only one person in the age group of 18–49 was selected from the “18-49 households.” Interviews were conducted face-to-face using a standardized interviewer training set, translation protocols, and a survey instrument.

The household questionnaire consisted of five sections, including a roster and sections about income, dwelling, transfers, expenditures, and assets. The individual questionnaire included sections on disability, health and its determinants, risk factors, work history, chronic conditions, subjective well-being, care-giving, health system responsiveness, and health care utilization (Kowal et al., 2012). A proxy questionnaire gathered information about functioning, chronic conditions, health, and healthcare utilization. The study also included a verbal autopsy module to determine the expected reason for deaths that occurred in the household in the 24 months preceding the interview. Person-level and household-level analysis weights were estimated, and post-stratification correction methods were used based on the latest population estimates.

The study aimed to examine the impact of widowhood on social capital among older female adults, comparing the different components of social capital between currently married and widowed older female adults. The analytical sample comprises older female adults who are either widowed or currently married.

### Methods

#### Outcome variable: selected components of social capital

The outcomes of interest for this study are selected components of social capital such as *sociability; trust and solidarity;* and *safety*. The SAGE survey covered a detailed perspective of social capital of the sampled population. The social capital promotes the well-being of all its members, fights exclusion and marginalization, creates a sense of belonging, promotes trust, and offers its members the opportunity of upward mobility (OECD, 2012). As mentioned earlier, the analytical sample (3,205) of this study is comprised of only female older adults of age 50 years and over. Outcome variables such as *sociability; trust and solidarity;* and *safety* include multiple domains.

The study employed a systematic approach to variable coding and transformation to ensure consistent measurement of social capital components. For sociability, responses were coded on a five-point scale from 1 (“never”) to 5 (“daily”), maintaining the original directionality where higher scores indicate greater social engagement.

For trust and solidarity measures, we recoded the original five-point scale to ensure consistent directionality with other social capital components. While the original SAGE questionnaire coded responses from 1 (“to a very great extent”) to 5 (“to a very small extent”), we reversed this coding so that higher scores consistently indicate greater trust levels. Similar recoding was applied to safety measures, ensuring that higher scores represent greater perceived safety.

The selection of control variables was guided by both theoretical frameworks and empirical evidence from previous studies on social capital and widowhood in India. Variables such as age, education, work status, household structure, and economic status were included based on their established relationships with both widowhood experiences and social capital formation in the Indian context (Samanta, 2020; Sahoo, 2014; Heath, 2014; Park et al., 2024). This selection ensures comprehensive control for socioeconomic and demographic factors that may influence the relationship between widowhood and social capital.

The measures of sociability included questions about how often participants engaged in a particular social activity in the last 12 months, including—attendance at a public meeting discussing local affairs; personally meeting a community leader; attending any group meeting (club, union, society, organization); working with other people in the neighborhood to improve or fix something; having friends visit their home; being in the home of or hosting someone from a different neighborhood; socializing with co-workers outside work; attending religious services and; leaving the house to attend meetings, activities, visit family or friends. The subject responded to each item according to the response options “never”, “once or twice per year”, “once or twice per month”, “once or twice per week” or “daily”, coded numerically from 1 (“never”) to 5 (“daily”).

Trust and solidarity were assessed with three items, (1) first think about people in your neighborhood. Generally speaking, would you say that you can trust them? (2) Now, think about people whom you work with. Generally speaking would you say that you can trust them? (3) And how about strangers? Generally speaking would you say that you can trust them? The subject responded to each item according to the response options “to a very great extent”, “to a great extent”, “neither great nor small extent”, “to a small extent” or “to a very small extent”, coded numerically from 1 (“to a very great extent”) to 5 (“to a very small extent”). While co-worker trust may appear more relevant for currently employed individuals, our approach considers the cumulative nature of social capital, where past workplace experiences contribute to an individual's overall trust disposition. This is particularly relevant in the Indian context, where informal work networks often persist beyond active employment.

Safety was assessed with 2 items (1) “In general, how safe from crime and violence do you feel when you are alone at home?” (2) How safe do you feel when walking down your street alone after dark? The subject responded to each item according to the response options “completely safe”, “very safe”, “moderately safe”, “slightly safe” or “not safe at all”, coded numerically from 1 (“completely safe”) to 5 (“not safe at all”).

Sociability score, safety score and trust and solidarity score have been generated for each female older adult covered in the survey by using an Item Response Theory (IRT) Partial Credit Model (PCM). The description of the method (IRT PCM) has been provided below and has been adopted from Zheng and Rabe-Hesketh (2007, p. 314–315) (kindly see the above paper for details; the notations have been retained for coherence and ease of readers).

The PCM is an extension of the Rasch model to polytomous items with ordered response categories 1, …………, 5 (it is 1 to 5 because in our case we have five response categories) for an item (or a question) i. The PCM specifies the probability of responding in the jth category of item i for person n as a function of the person ability *O*_*n*_ and step parameters δ_*ij*_ (*j* > 1).


(1) $$\begin{array}{l} Pr\left(x_{in}=j|\theta_{n}\right)=\frac{\exp\sum_{l=1}^{j}\left(O_{n}-\delta_{il}\right)}{\sum_{k=1}^{m_{i}}\exp\sum_{l=1}^{k}\left(O_{n}-\delta_{il}\right)}\;j=1,\;\ldots \ldots \;5 \end{array}$$

Where, $\sum_{l=1}^{1}\left(O_{n}-\delta_{il}\right)=0$. This is a special case of a multinomial logit model, namely, an adjacent category logit model (Agresti and Coull, 2002) with


(2) $$\begin{array}{l} \ln\frac{\mathrm{Pr}\left(x_{in}=j|O_{n}\right)}{\mathrm{Pr}\left(x_{in}=j-1|O_{n}\right)}=O_{n}-\delta_{ij} \end{array}$$

The parameter δ_*ij*_ is known as the step difficulty associated with category j of item i. It represents the added difficulty when moving the step from category j−1 to category j (Wilson, 2023). A two parameter logit (2PL) PCM (Muraki, 1992) can also be specified by including a slope parameter, λ_*i*_, that allows each item to have a different discrimination.

In the PCM, the linear predictors *v*_*ijn*_ represent the logarithm of the numerators of the response probabilities:


(3) $$\begin{array}{l} Pr\left(x_{in}=j|\theta_{n}\right)=\frac{exp\left(v_{ijn}\right)}{\sum_{k=1}^{m_{i}}exp\left(v_{ikn}\right)}\;j=1,\;\ldots \ldots \ldots \ldots ,\;5 \end{array}$$

Using multiple items (for measuring sociability, safety and trust and solidarity), five response categories to each item and the above discussed IRTPCM method (which uses the multiple items and multiple response categories to each item), scores (continuous) have been generated for each female older adult for aforementioned three components of social capital. Furthermore, these scores range from 0 (lowest sociability, lowest safety and lowest trust and solidarity) to 100 (highest sociability, highest safety and highest trust and solidarity).

#### Main independent and control variables

In this study, marital status is the main independent variable of interest. It has been captured as a categorical variable with the categories being currently married and widowed (as mentioned in the subsection 2.1, the sample comprises of only older female adults who were either widowed or currently married). There is a growing body of literature that indicates that in addition to widowhood, several other socioeconomic and demographic variables have substantial sway on the selected components of social capital. Therefore, the present analysis is also adjusted for multiple pertinent socioeconomic and demographic predictors of social capital in the analysis, such as age (categorized into three categories—50–59, 60–69 and 70+ years); schooling (categorized into four categories – no schooling, up to primary, above primary to secondary and above secondary); place of residence (categorized into two categories—rural and urban); work status (categorized into three categories—never worked, currently working, currently not working); household structure (categorized into two categories—nuclear and non-nuclear); religion (categorized into three categories—Hindu, Muslim and others); caste (categorized into three categories—SC, ST and others); and economic status of household (captured by wealth index).

### Statistical analysis

First, we have described the selected demographic and socioeconomic characteristics of the sample. Then we have calculated the prevalence of widowhood by selected socioeconomic and demographic characteristics. Thirdly, we have presented mean sociability score (MSOCS), mean safety score (MSAFS) and mean trust and solidarity score (MTS) by various socioeconomic and demographic variables for the older female adults. It is followed by ordered logistic regression models which have been estimated to assess the effects of widowhood on social capital after adjusting for socioeconomic, demographic and cultural characteristics. The ordered logistic regression models have been estimated separately for the three selected components of social capital—(1) sociability, (2) trust and solidarity and (3) safety. For the ordered logistic regression modeling, we have divided the sociability (1), trust and solidarity (2) and safety (3) scores (which were generated using IRTPCM technique mentioned earlier) equally into four categories (above 75 and up to 100 [highest]; above 50 and up to 75; above 25 and up to 50; greater than or equal to 0 and up to 25 [lowest]). Finally, we have computed predicted probabilities of being in the highest category (highest category refers to the topmost category with scores above 75 and up to 100) of sociability, safety, and trust and solidarity for the currently married and widowed population.

## Results

### Socioeconomic and demographic profile of female older adults

Table 1 presents the socioeconomic and demographic profiles of female older adults in India. The total number of female older adults in the sample is 3205. Around half of the sample population (henceforth, the population refers to female older adults aged 50 and over) belong to the 50–59 age group. Only 20% of the population are of 70 years or older in the study sample. A significant proportion (37%) of the sampled population is widowed. Approximately every third woman aged 50 and over is a widow. Surprisingly, almost 75% of the population does not have any exposure to formal education. Merely 3% of the female older adults could attain a 10 or more years of schooling. Almost half of the female older adults never did any paid work in their life. Only 20% of the female older adults are currently engaged in some kind of paid work. About 30% of the population is currently not working who were earlier working. The sample population is well distributed across different wealth quintiles. There is a striking gap in the proportion of the population living in urban and rural areas. Just 30% of the sample population habitats in urban areas and the rest of the population live in rural areas. Astonishingly a huge proportion of the population is enjoying a non-nuclear family structure and it is almost 80%. The majority of the sampled population belongs to the Hindu religion (85%) and 12% population is practicing Islam religion. As Indian society is visibly segregated by caste, which also forms the social fabric of the country, it becomes inevitable to understand the population distribution by caste. Almost 16% of the population belongs to Scheduled Caste (SC) group who forms the lowest socio-economic strata and is significantly lagging in socioeconomic indicators as compared to the population belonging to the other caste groups. About 5% of the sampled population belongs to the Scheduled Tribes (ST) which also contributes to the lowest socioeconomic groups in the country.

**Table 1:** Selected demographic and socioeconomic characteristics of female population aged 50 and over in India.

**Variables**	**India**
**Sample size**, ***N***	**3,205**
	(%)
**Age**	
50–59	47.82
60–69	32.02
70+	20.16
**Marital status**	
Currently married	62.60
Widowed	37.40
**Educational attainment**	
No formal education	73.24
Up to primary	15.57
6–10	8.45
10+	2.74
**Work status**	
Never worked	52.30
Currently working	20.52
Currently not working	27.18
**Wealth quintile**	
1	18.82
2	19.96
3	19.22
4	18.37
5	23.63
**Residence**	
Urban	29.51
Rural	70.49
**Household structure**	
Nuclear	19.79
Non-nuclear	80.21
**Religion**	
Hindu	84.59
Muslim	12.03
Other	3.39
**Caste**	
SC	16.19
ST	5.51
Other	78.30

Source: Authors' computation based on the Wave1 data of the SAGE Study on Global Aging and Adult Health (2007–08).

Wealth quintile 1 and 5 refer to the lowest and highest wealth group respectively; SC refers to Scheduled Caste; ST refers to Scheduled Tribe.

### Socioeconomic and demographic profile of widowed female adults

Table 2 shows the profile of widowed female older adults by different socioeconomic and demographic characteristics. As it can be seen, a substantially high proportion of female older adults are widows. Overall, 37% of the sample population are widowed, which is a big challenge for Indian society where sufficient social security for widows is generally lacking. As averages hide variation, we further tried to calculate the prevalence of widowhood by selected socioeconomic and demographic characteristics. The prevalence of widowhood proliferates as age advances. In the age group 50–59, 18% of the population are widows, whereas in the age group 60–69 it reaches 41%. Among the 70+ population, this proportion is enormously high and is about 75%. The extent of widowhood is the highest among the population who do not have any exposure to formal education (41%) and it is lowest among those who have 10+ years of formal education (17%). The prevalence of widowhood is 30% among those who are currently working while it is 45% among the population who are currently not engaged in any paid work. There is an inverse relationship between economic status and the incidence of widowhood. The poorest population has the highest prevalence of widowhood (50%). The wealthiest population experiences the least incidence of widowhood (29%) as compared to the poorest population (50%). There is no significant difference in the prevalence of widowhood by place of residence. The prevalence of widowhood is 39% in urban areas while it is 37% in rural areas. The analysis indicates that there is a remarkable difference in the prevalence of widowhood by family structure. The non-nuclear families experience four times higher (80%) prevalence of widowhood as compared to nuclear families (20%). The prevalence of widowhood is almost equal (37%) in two major religions of India, whereas it is significantly higher (53%) among the population who are practicing any religion other than Hinduism and Islam. If we see by caste, we observe that scheduled caste (SC) which are often referred to as “Dalits” or “lower caste”, have the highest prevalence of widowhood as compared to the remaining caste groups.

**Table 2:** Prevalence of widowhood by socioeconomic and demographic characteristics in India.

**Variables**	**Total sample (3,205)**
Overall	37.4
**Age**	
50–59	18.55
60–69	41.29
70+	75.89
**Educational attainment**	
No formal education	41.10
Up to primary	31.29
Above primary to secondary	23.03
Above secondary	17.40
**Work status**	
Never worked	35.79
Currently working	30.33
Currently not working	45.82
**Wealth quintile**	
1	50.02
2	37.26
3	40.07
4	32.35
5	29.09
**Residence**	
Urban	39.19
Rural	36.64
**Household structure**	
Nuclear	20.16
Non-nuclear	79.84
**Religion**	
Hindu	36.84
Muslim	36.88
Other	53.14
**Caste**	
SC	43.06
ST	36.35
Other	36.30

Source: Authors' computation based on the Wave1 data of the SAGE Study on Global Aging and Adult Health (2007–08).

Wealth quintile 1 and 5 refer to the lowest and highest wealth group respectively; SC refers to Scheduled Caste; ST refers to Scheduled Tribe.

### Mean sociability, trust and solidarity, and safety scores by socioeconomic characteristics

In Table 3, we have calculated the extent of three components of social capital, namely sociability; trust and solidarity; and safety among the currently married and widowed population. From Table 3, it is obvious that the currently married population is enjoying more sociability as compared to the widowed population. The mean sociability score (MSOCS) among the currently married population is 31, while it is only 26 among the widowed population. The difference in MSOCS among the currently married and widowed population is least (0.5 points) in the age group 50–59 while it is highest (6 points) for the population with 70+ age. There are notable differences in MSOCS among the currently married and widowed population across the different categories of education. The difference in MSOCS in the currently not working population among currently married and widowed is highest as compared to the remaining occupational categories. There is a positive relationship between MSOCS and wealth quintile among the currently married population. The difference in MSOCS among the currently married and widowed narrows down as the economic status increases. The difference in MSOCS among currently married and widowed population is higher in urban areas (6 points) as compared to rural areas (4 points). There is a strikingly large difference in MSOCS among the currently married and widowed population in non-nuclear families, which is four points. It must be noted that we have discussed in the last table that prevalence of widowhood is highest in the other religion category, but surprisingly the difference in MSOCS among the currently married and widowed population is almost negligible in this category of “other” religion. Among the caste categories, the highest difference in MSOCS between currently married and widowed has been observed among the “SC” group.

**Table 3:** Components of social capital among currently married and widowed females by socioeconomic and demographic characteristics in India.

**Variables**	**Sociability**		**Trust and solidarity**		**Safety**	
	**Currently married**	**Widowed**	**Currently married**	**Widowed**	**Currently married**	**Widowed**
Overall	30.8	26.1	43.8	42.4	60.5	59.8
**Age**						
50–59	31.5	32.0	44.8	42.9	59.4	61.0
60–69	29.9	25.9	42.8	44.4	61.5	61.9
70+	28.6	22.9	39.2	40.4	65.6	57.3
**Educational attainment**						
No formal education	31.0	26.0	44.6	42.9	61.0	59.7
Up to primary	30.9	27.1	41.9	40.3	57.4	57.0
Above primary to secondary	28.5	26.1	40.7	39.6	60.4	68.1
Above secondary	32.8	24.9	45.3	41.3	65.4	61.2
**Work status**						
Never worked	28.4	23.7	44.7	43.1	64.9	62.7
Currently working	35.4	33.8	43.9	43.3	55.1	56.2
Currently not working	31.8	25.9	41.6	40.8	55.7	57.3
**Wealth quintile**						
1	29.0	24.9	43.6	43.7	57.8	56.6
2	30.3	24.1	43.5	44.0	62.4	62.8
3	30.6	27.5	46.1	38.5	57.4	60.2
4	31.6	28.4	42.8	39.9	59.8	58.9
5	31.6	26.2	43.1	45.1	63.2	61.5
**Residence**						
Urban	29.6	23.7	43.0	41.4	57.6	52.3
Rural	31.3	27.2	44.1	42.8	61.7	63.2
**Household structure**						
Nuclear	31.4	29.0	43.0	43.7	57.9	60.3
Non-nuclear	30.6	25.6	44.0	42.2	61.3	59.7
**Religion**						
Hindu	30.8	25.9	43.2	42.1	60.8	59.3
Muslim	30.7	25.1	48.9	47.5	60.6	63.7
Other	32.0	31.9	40.2	34.0	51.5	59.1
**Caste**						
SC	30.3	24.7	43.6	39.9	61.6	57.5
ST	28.2	32.5	41.1	38.6	55.7	61.6
Other	31.1	26.0	44.0	43.2	60.6	60.3

Source: Authors' computation based on the Wave1 data of the SAGE Study on Global Aging and Adult Health (2007–08).

Wealth quintile 1 and 5 refer to the lowest and highest wealth group respectively; SC refers to Scheduled Caste; ST refers to Scheduled Tribe. All scores range from 0-100, with higher scores indicating greater levels of the respective social capital component. For sociability, higher scores represent more frequent social interactions; for trust and solidarity, higher scores indicate greater trust levels; for safety, higher scores represent higher perceived safety levels.

Table 3 also portrays the extent of trust and solidarity by socioeconomic and demographic characteristics. As it can be seen mean trust and solidarity score (MTS) is two points higher among the currently married population as compared to the widowed population in the study sample. The currently married population is experiencing higher MTS in contrast to the widowed population across all categories of selected socioeconomic and demographic characteristics except “age” and “household structure”. As age advances, the degree of trust and solidarity decreases among currently married female older adults. Across the categories of educational attainment, MTS is not showing a linear relationship. MTS is higher in two extreme categories of educational attainment than the middle categories of the same for both currently married as well as widowed. MTS is higher among the currently married population contrary to the widowed population for all categories of “work statuses”. In the 3rd wealth quintile, MTS is seven points higher among the currently married population in contrast to the widowed population. Those who are practicing the religion other than Hinduism and Islam, among them the difference in MTS between currently married and widowed population is highest (6 points).

Furthermore, Table 3 compares the mean safety score (MSAFS) between the currently married and widowed population across the pertinent socioeconomic and demographic variables. MSAFS is nine points less among the widowed population as compared to the currently married population in the 70+ age group. Similarly, among the population who have 10+ years of education, MSAFS is four points lower among the widowed population in comparison with the currently married population. Among the urban habitats, MSAFS among the currently married population is 58 while it is only 52 among the widowed population. In the SC category of caste, MSAFS is four points higher among the currently married population in contrast to the widowed population.

### Multivariate analysis: ordered logistic modeling of sociability, trust and solidarity, and safety among older female adults

Table 4 provides the proportional odds ratios for the ordered logistic model of responding in any higher category of sociability, trust and solidarity, and safety among female older adults of India by selected socioeconomic and demographic characteristics. We will discuss the proportional odds ratios separately for the selected components of social capital, that is: sociability, trust and solidarity, and safety. The odds of being in any higher level of sociability is 17% (significant at *p* < 0.05) lesser for widowed than currently married population. The 70+ population is having 45% (significant at *p* < 0.01) lesser chance to achieve any higher level of sociability as compared to the female older adults of the age group 50–59. Those who are participating in the labor force are having 2.5 times higher chances of being highly socialized in contrast to those who never engage in paid work. Female older adults of top wealth quintile have 75% chances to enjoy higher sociability in comparison with the lowest wealth quintile population. Similarly, the population who is living in rural areas is 1.5 times more likely to adore the advanced level of sociability than their urban counterparts.

**Table 4:** Ordered logistic regression model estimating effects of marital status along with socioeconomic and demographic characteristics on components of social capital in India.

**Variables**	**Sociability**	**Trust and solidarity**	**Safety**
**Marital status**			
**Currently married** ^®^			
Widowed	0.83^**^	0.86^**^	1.12
**Age**			
**50–59** ^®^			
60–69	0.89	0.86^**^	0.99
70+	0.55^***^	0.62^***^	1.03
**Educational attainment**			
**No formal education** ^®^			
Up to Primary	0.91	0.86	0.98
Above primary to secondary	1.08	0.94	1.02
Above secondary	1.07	1.04	1.48^**^
**Work status**			
**Never worked** ^®^			
Currently working	2.54^***^	2.18^***^	0.72^***^
Currently not working	1.40^***^	1.26^***^	0.70^***^
**Wealth quintile**			
**1** ^®^			
2	1.140	1.090	0.98
3	1.41^***^	1.31^**^	1.02
4	1.43^***^	1.37^***^	1.01
5	1.73^***^	1.53^***^	1.22
**Residence**			
**Urban** ^®^			
Rural	1.51^***^	1.69^***^	1.20^**^
**Household structure**			
**Nuclear** ^®^			
Non-nuclear	0.96	1.07	0.97
**Religion**			
**Hindu** ^®^			
Muslim	0.85	0.86	1.01
Other	1.07	0.86	0.62^**^
**Caste**			
**SC** ^®^			
ST	1.04	1.06	0.79
Other	1.03	0.96	1.03

Source: Authors' computation based on the Wave1 data of the SAGE Study on Global Aging and Adult Health (2007–08).

Wealth quintile 1 and 5 refer to the lowest and highest wealth group respectively; SC refers to Scheduled Caste; ST refers to Scheduled Tribe. ^®^−Reference Category; ^***^p < 0.01, ^**^p < 0.05, ^*^p < 0.10.

Table 4 also portrays the proportional odds ratios for the trust and solidarity component of social capital. The probability of having a higher level of trust and solidarity among the widowed population is 14% (significant at *p* < 0.05) less as compared to the currently married population. Similarly, the population belonging to the age group 60–69 are also having 14% lesser chances of having high trust and solidarity than those of the 50–59 age group. The oldest old (70+) population is almost having 40% fewer chances of enjoying a higher trust and solidarity than the population of the 50–59 age group. The working older adults are having 2.2 times higher chances of relishing adequate trust and solidarity in society than those older adults who never worked. The richest population is 50% more likely to cherish sufficient trust and solidarity than the poorest population. Likewise, rural residents are having 70% (significant at *p* < 0.01) higher likelihood of enjoying a higher level of trust and solidarity as compared to their urban counterparts. The results for the safety component of social capital are inconclusive, and hence not been discussed here.

Analysis of residential patterns reveals significant variations in social capital components between urban and rural settings. Rural residents demonstrated notably higher sociability scores (31.3 among currently married and 27.2 among widowed) compared to their urban counterparts (29.6 among currently married and 23.7 among widowed). This urban-rural difference was particularly pronounced among widowed women, suggesting that rural settings may offer more opportunities for social interaction and community engagement. Similarly, trust and solidarity scores showed stronger preservation in rural areas, with rural widows maintaining higher scores (42.8) compared to urban widows (41.4).

### Predicted probabilities of enjoying the highest level of social capital

**Figure 1:**
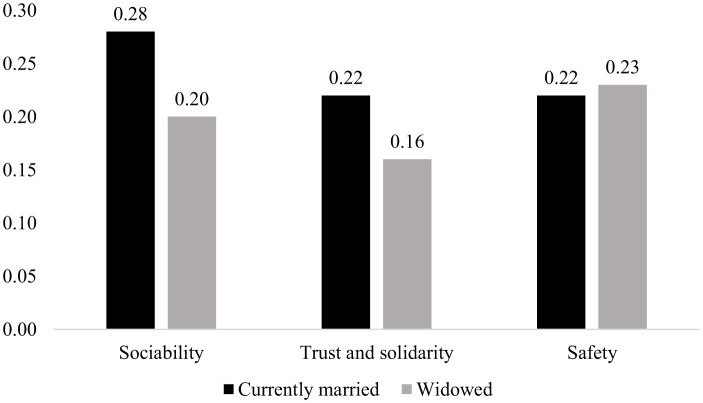
Probability of responding in the highest category of different components of social capital by marital status in India.

Figure 1 shows the predicted probability of being in the highest category of different components of social capital by marital status in India. It is clear from the figure that the widowed population is having an enormously low probability of being in the top category of “sociability” and “trust and solidarity” with respect to the currently married population. The widowed population has eight percent fewer chances of adoring the highest level of sociability with respect to the currently married population. The predicted probability of having the highest level of trust and solidarity among the currently married population is 0.22, while it is only 0.16 for the widowed population. However, there is no significant difference in the “safety” component.

## Discussion

Widowhood possibly is the most tragic event in the life of a woman, which increases her vulnerability as she grows older (Bagchi, 1998). Social capital plays a crucial role in achieving a better quality of life and material wellbeing, particularly among older adults. Components of social capital like ‘trust and solidarity' and ‘sociability' have positive effects on the well-being of individuals, particularly among societies with rigid cultural and social environments. A few research attempts have been made so far to explore the well-being of widows from both the socio economic and health perspective at global and country specific levels (Kertzer and Laslett, 1995). However, there is still a paucity of empirically rigorous work to understand the impact of widowhood on social vulnerability, loss of trust and sociability. The present study perhaps is the first attempt to comprehensively and empirically examine the relationship between widowhood and social capital in the Indian context.

Existing studies have documented that better socio-economic conditions are positively associated with social viability and social capital, particularly at older ages (Agren, 2004). Financial securities also play a significant role in good health and social wellbeing (Carman et al., 2003). While examining the socio-demographic factors, results from the current study suggest that factors like age, marital status, household structure, ethnicity and wealth status play vital role in determining the levels of social capital like trust, solidarity and sociability. The social capital of widows is also affected by educational status and geographical location. With globalization and improvement in the standard of living, it is generally perceived that social capital has improved in the developing countries resulting in lower levels of marginalization and vulnerability. However, our study shows that deprivation and vulnerability associated with widowhood in older ages is still highly prevalent across India. Widows in India face significantly lower levels of sociability and solidarity. The impact of widowhood is apparent across all the socio-economic settings and is in line with existing studies (Elwert and Christakis, 2006; Lee et al., 2001).

Researchers even manifest that the life of a widow ends up in isolation, loneliness and distress with increasing the risk of morbidity and other short term health issues (Owen, 1996). These studies have highlighted the role of socio-economic opportunities which are mostly lacking for widows which make them more vulnerable than married females (Wardle et al., 2002; Manzoli et al., 2007). This is supported by our findings as well. That is, older females who are participating in the labor force are having 2.5 times higher chances of being highly socialized in contrast to those who never engage in paid work. Similarly, the working older adults are having 2.2 times higher chances of relishing adequate trust and solidarity in society than those older adults who never worked.

As we found that the older widows in India experience lesser sociability, trust and solidarity, this can be attributed to various reasons. The patriarchal structure prevailing in India over the years has resulted in social isolation and economic discrimination of widows thus making them more vulnerable (Dreze, 1990). Gender based violence has also contributed significantly to the loss of fundamental human rights and eventually leading to loss of social dignity among the widows (Hollstein, 1998). Moreover, the social condition of widows in India has further worsened because of religious symbolism and rigid cultural norms which are still rampant in Indian societies. Our findings also reveal important urban-rural differences in social capital formation among older widows in India. The higher sociability observed in rural areas (51% higher than urban areas) suggests that traditional community structures and social networks may be more preserved in rural settings, potentially buffering some negative effects of widowhood. However, this urban-rural divide warrants further investigation, particularly as urbanization continues to reshape social relationships and support systems for older adults in India.

Our study has a few strengths, such as it rigorously (both comprehensively as well as empirically) examines the relationship between widowhood and social capital in the Indian context; it is based on data from population-based nationally representative sample survey to analyse the linkage between widowhood and various aspects of social capital, population-based large scale survey have advantage over case-based studies in terms of their representativeness of the population as a whole; also, our study uses a comprehensive and holistic conceptualization of social capital; the framework of social capital which has been used in our study includes multiple domains; where the domains themselves comprise of several items. Having mentioned the advantages, this study also has some limitations: the sample was taken from the six populous states of India namely Assam, Karnataka, Maharashtra, Rajasthan, Uttar Pradesh and West Bengal; though these states cover all the regions of India (West Bengal from East, Maharashtra from West, Rajasthan and Uttar Pradesh from North, Karnataka from South and Assam from North-East), there could still be some interstate variations which we could not capture. There can also be some apprehension about the comprehensive nature of social capital as captured in our study. We cannot rule out an even more comprehensive conceptualization of social capital in the Indian context.

## Conclusion and policy suggestions

The social, legal, economic and cultural situation of the Indian widows calls for an urgent response at all levels of society. This urgency is augmented by the fact that, in all parts of the country, the number of widows is substantial because of the significant age difference between the spouses and the higher life expectancy of females. Hence, the aging pattern indicates that an overwhelmingly large proportion of the geriatric population in the country will be females; a large proportion among them will be widows who require support (The Global Widows Report, 2015). Thus, external bodies, such as UN and other international agencies need to influence and support the Government of India in taking up programs for the welfare of widows as the issue remains a low priority on the policy agenda of the country.

Existing literature describes different strategies like independent household, remarriage, living with children, becoming an inmate or migration for improving the welfare of widows. However, these strategies are mostly determined by the circumstances and economic rights assigned to a widow (Dribe et al., 2006). Thus, policies should aim at empowering women in general and widows in particular so that they can help themselves to mitigate the widowhood challenges. An inclusive approach could also include a reasonable amount of widow pension for older widows who are unable to take care of their expenses (Bíró, 2013). Though widow pension schemes exist in India, the amount paid and level of implementation both are poor.

Social capital is one of the keys to overcome the challenges of widowhood vulnerability, particularly among older females. That can be done through social networks, social support and better social inclusiveness (United Nations, 2001). Further, it will help if widows can be provided space within Self Help Groups (SHGs), which will provide them with a higher chance of having sufficient social capital. This could be important because the existence of SHGs is widespread across the country and they are involved in various social and economic activities; studies have even highlighted the fact that better opportunities along with social inclusion, can uplift the life of widows, particularly in rural India, where women are extremely vulnerable (Chen, 1997).

Moreover, policymakers need to launch mass sensitization programs and campaigns to make the society aware of the situations and needs of widowed older adults. The widowed older population should themselves be involved in the discussion and dialogue for the policymaking. According to UNHCR (2011), there should be the active involvement of widowed older adults in all matters concerning them in the process of forming policies and programs. Health related policies must also focus on widowed older adults, with an attempt to integrate social, physical and public health domains.

Furthermore, existing research clearly indicates that widowhood not only leads to deprivation among widows but also in their families and children (Chen, 1997). It is found that children of widows also suffer multiple deprivations and are at higher risk of vulnerability (JISC, 2009). Therefore, it has been argued that policies like stronger laws on inheritance; registration of property both in the wife's and husband's names; automatic transferal of property or land to the widow upon husband's death; preference to widows in the schemes of land distribution; mandatory marriage registration; and affirmative action to stop the drop out of the widows' children from school can contribute to improved welfare of widowed older adults and their families. Last but not least, a review of the pension scheme in terms of both value, as well as implementation, can help in uplifting the position of widows in India (United Nations, 2001).

### Limitations

While the cross-sectional analysis in the study provides valuable insights into associations between widowhood and social capital, it cannot fully disentangle potential mediating pathways through variables like work status and household structure. Future longitudinal studies would be valuable to examine how these factors may mediate the relationship between widowhood and social capital development over time, particularly in the Indian context where both family structures and women's work participation are undergoing significant changes.

Besides, while we consider community safety as a factor that can facilitate social interactions and contribute to the accumulation of social capital (United Nations Development Programme, 2009; Elmasri, 2024; Rahman and Singh, 2019), we acknowledge that a significant body of literature conceptualizes safety primarily as an outcome rather than a component of social capital. The directionality of this relationship remains complex and context-dependent, necessitating caution in interpretation. Therefore, we urge readers to consider the nuanced interplay between safety and social capital, recognizing that while safer environments may foster greater social cohesion, pre-existing social capital can also contribute to community safety. This distinction is particularly relevant in the context of our study, and we highlight this as an area for further theoretical and empirical investigation.

## Data Availability

The datasets presented in this study can be found in online repositories. The names of the repository/repositories and accession number(s) can be found below: https://www.iipsindia.ac.in/sage.

## Footnotes

^1^End Notes The Vedic period, or Vedic age (c. 1500–c. 500 BCE), is the period in the history of the northern Indian subcontinent between the end of the urban Indus Valley Civilisation and a second urbanisation which began in the central Indo-Gangetic Plain c. 600 BCE (Roy, 2016).

^2^Smritis refer to Hindu scriptures. Manusmriti was one of the first Sanskrit texts to have been translated into English in 1794, by Sir William Jones, and was used to formulate the Hindu law by the British colonial government (Rani, 2017).

^3^Pertaining to Brahmins, which is the uppermost class in Hinduism who specialise as priests, teachers (acharya) and protectors of sacred learning across generations (Roy, 2016).
